# Supplementary material for: A Combined Deep Eutectic Solvent–Ionic Liquid Process for the Extraction and Separation of Platinum Group Metals (Pt, Pd, Rh)
Source: Molecules. 2021 Nov 27;26(23):7204. doi: 10.3390/molecules26237204 (PMC8659014; doi:10.3390/molecules26237204)
Supplement: Supplementary file 1 [file molecules-26-07204-s001.zip › molecules-1471259-supplementary.pdf]

A Combined Deep Eutectic Solvent–Ionic Liquid Process for the Extraction and Separation of Platinum Group Metals (Pt, Pd, Rh)

Olga Lanaridi,[a] Sonja Platzer,[a] Winfried Nischkauer,[b] Andreas Limbeck,[b] Michael Schnürch, and Katharina Bica-Schröder[a]

[a] Institute of Applied Synthetic Chemistry, Technical University of Vienna, Getreidemarkt 9/163, Vienna, Austria

[b] Institute of Chemical Technologies and Analytics, Technical University of Vienna, Getreidemarkt 9/164, Vienna, Austria

Supporting information contains five pages;

Supporting information contains three figures;

Supporting information contains two tables.

1. Synthesis of Phosphonium Ionic Liquids

1.1 Synthesis of P_{66614}B2EHP

P_{66614}Cl (51.91 g; 100.0 mmol) and bis(2-ethylhexyl) phosphate (B2EHP, 35.86 g; 111.2 mmol) were suspended in 100 mL of a mixture of H_2O:MeOH 1:1 and stirred for 2 h at RT. The solution was concentrated to approx. 60 mL and diluted with H_2O and repeatedly extracted with CH_2Cl_2. The combined organic layers were washed with H_2O until no more chloride ions could be detected (checked by the addition of aqueous $AgNO_3$ solution). The organic layer was collected and dried over Na_2SO_4 and concentrated under reduced pressure. The remaining solvent traces were removed under a high vacuum (0.01 mbar, 24 h), stirring at 50 °C to yield P66614B2EHP as a light yellow viscous liquid in 80% yield.

Analysis

^{1}H-NMR (400 MHz, Chloroform-*d*): δ (ppm) = 3.70 (dd, 4H), 2.39 m (8H), 1.47 (m, 20 H), 1.27-1.21 (m, 46H), 0.85 (m, 24H).

^{13}C-NMR (101 MHz, Chloroform-*d*): δ (ppm) = 67.04, 40.55, 32.00, 31.27, 30.94 (J_{P-H} = 15.0 Hz), 30.61 (J_{P-H} = 14.7 Hz), 30.20, 29.77, 29.73, 29.64, 29.44, 29.18, 23.42, 23.28, 22.78, 22.40, 22.06 (J_{P-H} = 4.6 Hz), 19.13 (J_{P-H} = 48.5 Hz), 14.24, 14.20, 14.04, 11.11.

^{31}P-NMR (162 MHz, DMSO-d_6) δ 33.76, -0.40.

ATR-IR v_{max} (cm^{-1}): 2955 (C-H stretch), 2923 (C-H stretch), 2854 (C-H stretch), 1467, 1379 (P=O stretch), 1246 (C-H bend), 1059 (C-H bend).

1.2 Synthesis of P_{66614}DOP

P_{66614}DOP was prepared according to the same procedure reported for P_{66614}B2EHP starting from P_{66614}Cl (51.93 g; 0.10 mol), bis(2,4,4-trimethylpentyl)phosphinic acid (57.13 g; 0.11 mol) and KOH (6.17 g; 0.11 mol). The product was obtained as a viscous liquid in 92% yield.

Analysis

^{1}H-NMR (400 MHz, DMSO-*d*$_6$) δ (ppm) = 2.18 (m, 8H), 2.02 (m, 2H), 1.53-1.18 (m, 52 H), 1.04-0.79 (m, 40H).

^{13}C-NMR (100 MHz, Chloroform-*d*) δ (ppm) = 54.40, 31.80, 31.30, 30.90 (J_{P-H} = 14.9 Hz), 30.39, 29.67, 29.64, 29.53, 29.34, 29.11, 25.78 (J_{P-H} = 10.0 Hz), 24.52, 22.68, 22.40, 22.07 (J_{P-H} = 5.0 Hz), 18.97 (J_{P-H} = 46.0 Hz), 14.10, 14.00.

^{31}P-NMR (162 MHz, DMSO-*d*$_6$) δ (ppm) = 33.75, 26.21.

ATR-IR v_{max} (cm^{-1}): 2955 (C-H stretch), 2952 (C-H stretch), 2857 (C-H stretch), 1466, 1362 (P=O stretch), 1172 (P=O stretch), 1050 (C-H bend), 1032 (P-O stretch).

2. Characterization of Car Catalyst Material

The spent car catalyst used in this study was fully characterized prior to the experiments (Figure 1). It was digested with the aid of a mixture of mineral acids in a microwave oven (Multiwave 3000, Anton Paar, Germany) prior to measurement, for the complete dissolution of the ceramic material, which primarily comprised the car catalyst. The following digestion program was employed: 8:00 at 500 W; hold 8:00; 15:00 at 900 W; hold 35:00. The quantification was performed by ICP–OES (Radial iCAP6500, Thermo Scientific, USA) with appropriate sample dilution. The fitness for purpose of the analytical measurement method was initially verified with the aid of the certified reference material ERM-EB504.

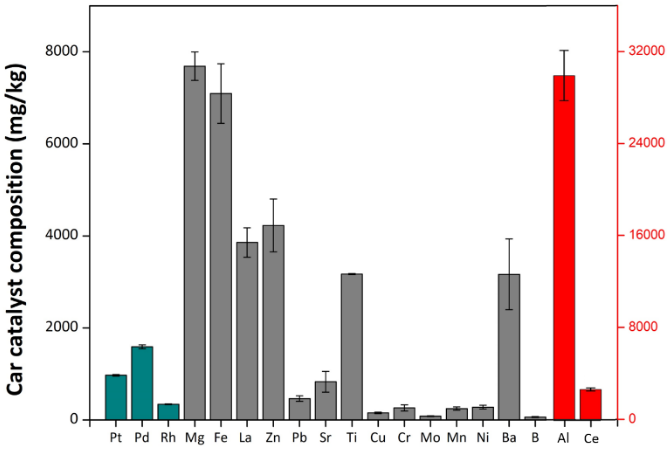

Figure S1. Concentration of elements present in the car catalyst material.

3. Leaching of PGMs from Spent Car Catalyst

The leaching process was performed as follows: A total of(0.1000 ± 0.0050) g ground car catalyst was mixed with (0.5000 ± 0.0100) g DES (solid: liquid 1:5) and (0.1000 ± 0.0050) g oxidizing agent (HNO_3), and the mixture was stirred at (80 ± 2) °C for 4 h. The mixture was subsequently centrifuged for 30 min at 13.500 rpm for the sedimentation of the solid car catalyst material. The liquid phase was recovered and appropriately diluted prior to PGM quantification by ICP–OES analysis. The leaching efficiency was calculated based on the following equation:

$$\%\, leaching\ efficiency = \frac{\mu g\ of\ [M]\ in\ the\ leachate}{\mu g\ of\ [M]\ in\ amount\ of\ catalyst\ powder\ (prior\ ro\ leaching)} * 100,$$

where [M]= metal

4. Extraction from Real Car Catalyst Sample

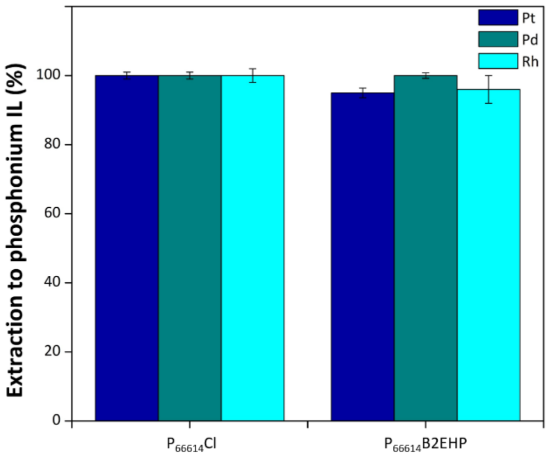

Figure S2. Extraction efficiencies (%) of PGMs from real autocatalyst leachate to $P_{66614}Cl$ and $P_{66614}B2EHP$.

5. Recycling of Hydrophilic IL-NMR Data

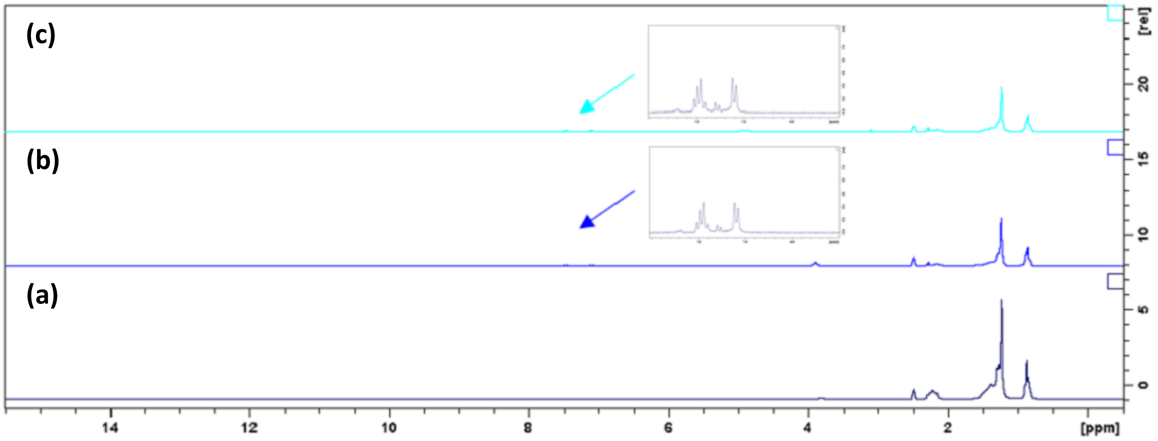

Figure S3. ^{1}H-NMR spectra of $P_{66614}Cl$ (a) pure, (b) after 1st cycle of L-L separation, and (c) after 2nd cycle of L–L separation, recorded on a Bruker Avance 200 MHz, DMSO-D_6, dilution 1:16, No of scans: 16.

6. ICP-OES Measurement Parameters

The PGMs concentrations in all systems were quantified with a radial ICP–OES (Thermo iCAP 6500, Thermo Scientific, USA) with a sample introduction kit consisting of a parallel path nebulizer (PEEK Mira Mist, Thermo Scientific, Canada), a gas cyclonic spray chamber with a riser tube, and a torch injector tube with 2 mm inner diameter. Signals in ICP–OES were recorded and processed using Qtegra 2.10 software (Thermo Scientific, USA). The detailed data on instrumental and measurement parameters are presented in Tables 1 and 2.

Table S1. ICP–OES instrumental measurement parameters.

**Instrument**	Radial iCAP 6500 (Thermo Scientific, USA)
**Software**	Qtegra 2.10 (Thermo Scientific, USA)
**RF power**	1400 W
**Torch internal diameter**	2.0 mm
**Nebulizer**	V-groove nebulizer (Thermo Scientific, USA)
**Nebulizer gas flow**	0.70 L/min
**Coolant gas flow**	12 L/min
**Auxiliary gas flow**	0.80 L/min
**Sample introduction flow rate**	0.70 mL/min
**Viewing height**	10 mm
**Exposure time (in Vis)**	10 sec
**Observation time per measurement**	5 sec
**Number of replicates premeasurement**	5

Table S2. Selected emission wavelengths for elemental quantification.

Element	Quantification wavelength	Quality control wavelength
Pt	265.945	214.423
Pd	340.458	324.270
Rh	343.489	369.236
Al	309.271	237.312
Fe	259.940	238.204
Zn	213.856	202.548
Sr	407.771	421.552
Mg	279.553	280.270
Ca	315.887	318.128
Pb	220.353	216.999
Ce	456.236	380.152
Cu	324.754	327.396
Ni	221.647	231.604
Mn	257.610	293.930
Cr	267.716	-
Ba	455.403	-
B	249.773	249.678
Mo	202.030	-
La	261.034	-
Ti	334.941	308.802
